# Revealing the Mechanism of *Astragali Radix* against Cancer-Related Fatigue by Network Pharmacology and Molecular Docking

**DOI:** 10.1155/2021/7075920

**Published:** 2021-12-08

**Authors:** Yi Xie, Kainan Zhou, Yan Wang, Shuhan Yang, Suying Liu, Xueqian Wang, Ying Zhang

**Affiliations:** ^1^Guang'anmen Hospital, China Academy of Chinese Medical Sciences, Beijing 100053, China; ^2^Beijing University of Chinese Medicine, Beijing 100029, China

## Abstract

**Background:**

Cancer-related fatigue (CRF) is an increasingly appreciated complication in cancer patients, which severely impairs their quality of life for a long time. *Astragali Radix* (AR) is a safe and effective treatment to improve CRF, but the related mechanistic studies are still limited.

**Objective:**

To systematically analyze the mechanism of AR against CRF by network pharmacology.

**Methods:**

TCMSP was searched to obtain the active compounds and targets of AR. The active compound-target (AC-T) network was established and exhibited by related visualization software. The GeneCards database was searched to acquire CRF targets, and the intersection targets with AR targets were used to make the Venny diagram. The protein-protein interaction (PPI) network of intersection targets was established, and further, the therapeutic core targets were selected by topological parameters. The selected core targets were uploaded to Metascape for GO and KEGG analysis. Finally, AutoDock Vina and PyMOL were employed for molecular docking validation.

**Results:**

16 active compounds of AR were obtained, such as quercetin, kaempferol, 7-O-methylisomucronulatol, formononetin, and isorhamnetin. 57 core targets were screened, such as AKT1, TP53, VEGFA, IL-6, and CASP3. KEGG analysis manifested that the core targets acted on various pathways, including 137 pathways such as TNF, IL-17, and the AGE-RAGE signaling pathway. Molecular docking demonstrated that active compounds docked well with the core targets.

**Conclusion:**

The mechanism of AR in treating CRF involves multiple targets and multiple pathways. The present study laid a theoretical foundation for the subsequent research and clinical application of AR and its extracts against CRF.

## 1. Introduction

Cancer-related fatigue (CRF) is a long-lasting physical, emotional, and/or cognitive tiredness or exhaustion associated with cancer or cancer treatment [1]. There is a huge distinction between CRF and other types of fatigue, which do not match the amount of recent activity, seriously interfere with body function, and even lead to the interruption of antitumor therapy. From 50% to 90% of cancer patients suffer from CRF during treatment or even after the end of treatment [2]. The study result indicates that 30%–60% of cancer patients develop moderate to severe fatigue in therapy that leads to the discontinuation of oncological treatment. In addition, more than one-fourth of patients still experience fatigue for more than 5 years after successful treatment, which seriously reduces the quality of life [3]. The development of CRF is mainly associated with inflammation, immune dysregulation, hypothalamic-pituitary-adrenal axis disturbances, and reduced energy metabolism [4]. Since the specific pathogenesis of CRF has not been stated clearly, clinical drugs are still in the exploratory stage. The current interventions are mainly exercise and targeted psychological and mental-body treatments, but the therapeutic effect is still not ideal [5].

*Astragali Radix* (AR) is the dried root of *Astragalus membranaceus* Bunge. As an effective and widely accepted treatment, AR has been applied to a variety of diseases. AR was first contained in the ancient Chinese herbal book Shennong Ben Cao Jing in the Han Dynasty and is listed as a good product for tonifying qi and tonifying deficiency. It was demonstrated that AR has immunomodulatory, antioxidant, and anti-inflammatory effects by modern pharmacological studies [6].

AR has a significant effect on CRF. Clinical practice shows that traditional Chinese medicine decoction and injection with AR, the main drug, can effectively improve the symptoms of CRF and is widely used in the treatment. Jeong et al. used multiple evaluation scales to prove that Buzhong Yiqi decoction with AR as the main drug can effectively alleviate the fatigue state of cancer patients [7]. Xu et al. found that Renshen Yangrong decoction containing a large amount of AR can significantly reduce the fatigue level of patients with moderate-to-severe CRF [8]. In addition, a multicenter, double-blind, randomized phase IV study showed that Astragalus polysaccharide injection can be used as an effective and safe treatment to relieve CRF [9].

Network pharmacology can map herbal compounds and disease phenotypes into biomolecular networks and conduct a complex network of disease-gene-target-drug through qualitative and quantitative analysis [10, 11]. Molecular docking calculates the matching degree of binding between molecular ligands and protein receptors, which is able to validate the analysis results of network pharmacology [12].

**Figure 1:**
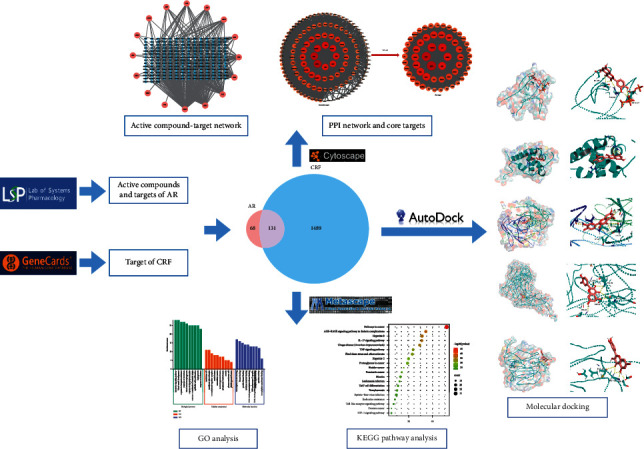
Workflow of the present research.

Consequently, this study made an elaborate investigation into the pharmacological mechanism of AR in treating CRF by network pharmacology (Figure 1). It furnishes a theoretical basis for the further investigation and clinical practice of AR against CRF. Firstly, the compounds and corresponding targets of AR were searched through TCMSP, and CRF targets were searched through the GeneCards database. AR targets were intersected with CRF targets to acquire the Venny diagram. The AC-T network was framed through Cytoscape 3.8.2. The STING platform was utilized to establish the PPI network. Then, according to the topological parameters, the core targets were extracted. Metascape was utilized to proceed GO and KEGG analysis. Finally, the molecular docking of compound ligands and target receptors was verified through AutoDock Vina.

## 2. Materials and Methods

### 2.1. Gathering of Active Compounds and Prediction of Targets

“Huangqi” (AR) was used as a search term in TCMSP [13] (http://tcmspw.com/tcmsp.php) to acquire related compounds. The aforementioned compounds that conformed to the criterion were selected and regarded as active compounds of AR: oral bioavailability (OB) ≥ 30% and drug likeness (DL) ≥ 0.18 [14]. The corresponding targets were extracted as AR targets. In order to prevent deviation caused by different naming methods, AR targets and subsequent CRF targets were normalized in the UniProt protein database [15] (https://www.uniprot.org).

### 2.2. Collection of CRF Targets

Using “cancer-related fatigue” as the keyword, the GeneCards database [16] (http://www.genecards.org) was searched to gain CRF targets. Through EXCEL software, the intersection of AR targets and CRF target was taken to draw a Venny diagram.

### 2.3. Establishment of AC-T Network

An AC-T network was established to display visually the intricate relationships between compounds and targets by the employment of Cytoscape 3.8.2 [17]. Network feature analysis was performed by the “Network Analyze” plugin to screen the main active compounds.

### 2.4. Establishment of PPI Network and Filtrating of Core Targets

The intersection targets were transmitted to the STRING platform [18] (https://string-db.org) to obtain PPI relationships. The CSV file containing the PPI relationship was downloaded to establish the PPI network. In this network, degree centrality (DC) determined the color depth and diameter size of the node. Targets with DC exceeding the average value were screened as core targets.

### 2.5. GO and KEGG Analysis

As an online tool for gene enrichment analysis, Metascape [19] (http://metascape.org/) integrated more than 40 gene function annotation databases. The core targets were uploaded into the Metascape platform to carry on Go and KEGG analysis. Data with *P* < 0.05 were selected as the analysis results.

### 2.6. Molecular Docking

Searching PDB database (http://www.rcsb.org) and TCMSP, structural files of the main active compounds as well as core targets were downloaded and saved as pdbqt format. The coordinate files of receptors and ligands were preprocessed by AutoDockTools 1.5.6 software and saved in pdbqt format: the water molecules in the ligands were removed, the ligands and receptors were separated, nonpolar hydrogen was added, and the Gasteiger charge was calculated. Through the gradient algorithm and multithreading technology of AutoDock Vina software, the docking activity between receptors and ligands can be evaluated by molecular docking score. Docking scores <−4.25, <−5.0, and <−7.0, respectively, represent the existence, good, and strong docking activity between the ligand and the receptor. The docking of molecular ligands and protein receptors was intuitively exhibited through PyMOL software.

## 3. Results

### 3.1. Compounds of AR and Intersection Targets

87 compounds of AR were extracted through TCMSP. 16 active compounds (Table 1) were selected through OB and DL parameters. Targets corresponding to active compounds were extracted and transmitted into EXCEL software, the repeated items were deleted, and a total of 199 AR targets were acquired after merging.

**Figure 2:**
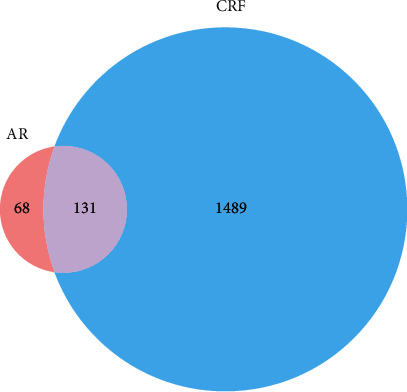
Venny diagram of AR targets and CRF targets (the area of the circle is proportional to the number of targets).

1620 CRF targets were collected through the GeneCards database. AR targets and CRF targets were mapped, resulting in a total of 131 intersection targets. The mapping result of AR targets and CRF targets was made into the Venny diagram (Figure 2).

### 3.2. AC-T Network

**Figure 3:**
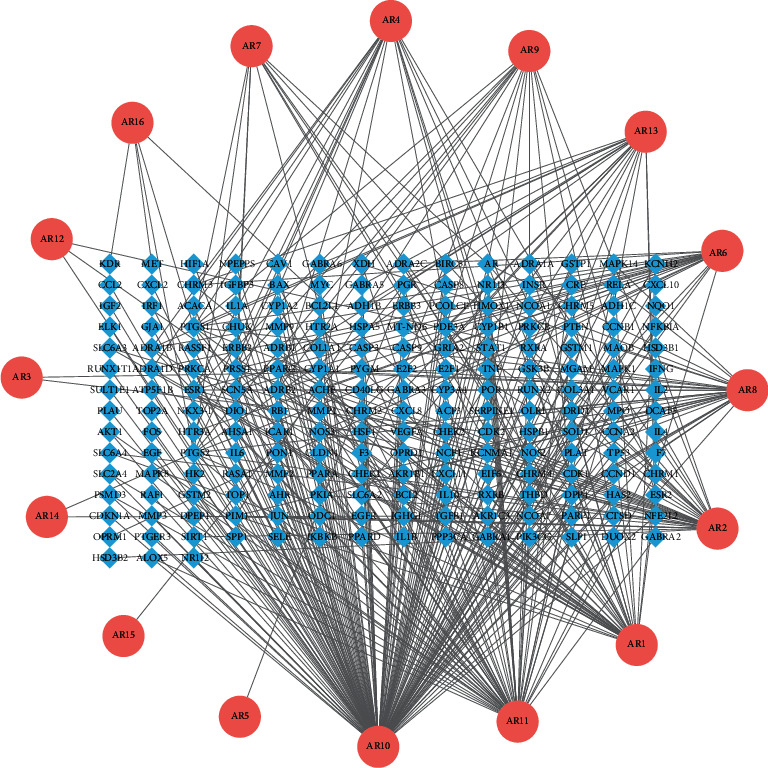
AC-T network.

In the AC-T network (Figure 3), the DC of a single target indicates the number of connected nodes. The network was analyzed by the “Network Analyze” plugin, and the active compounds were ranked by DC. The top five active compounds (Table 2) were set out with betweenness centrality (BC), closeness centrality (CC), and DC.

### 3.3. PPI Network and Core Targets

**Figure 4:**
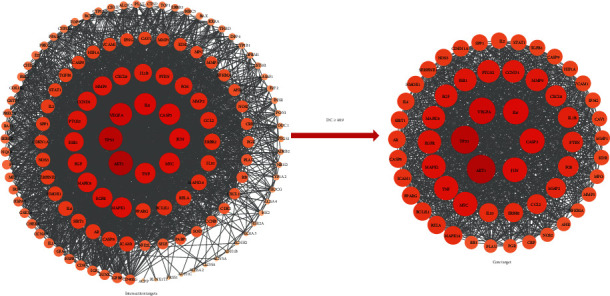
The PPI network of intersection targets and core targets (the larger the node, the darker the color, indicating the larger the DC).

There were 131 nodes and 2676 edges in the PPI network of intersection targets. Node size and color depth were proportional to DC. Through further analysis of the topological parameters, nodes meeting the criteria of DC ≥average value (40.9) were extracted. 57 core targets were screened out. The PPI network of intersection targets and core targets is displayed in Figure 4. Ranked by DC, the top fifteen core targets are listed in Table 3.

### 3.4. GO and KEGG Analysis

**Figure 5:**
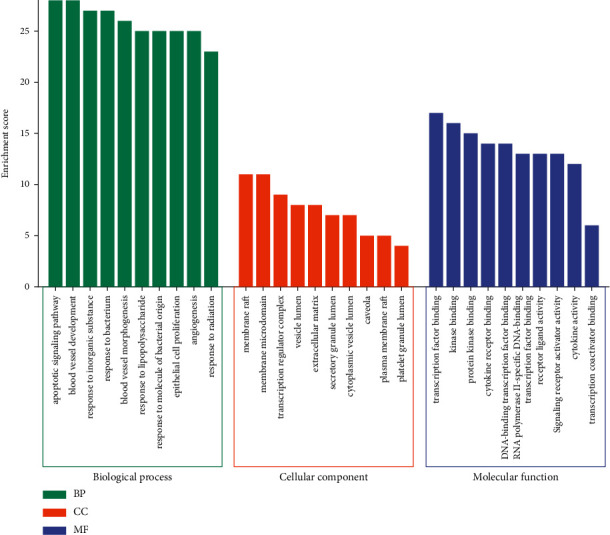
GO analysis of core targets.

**Figure 6:**
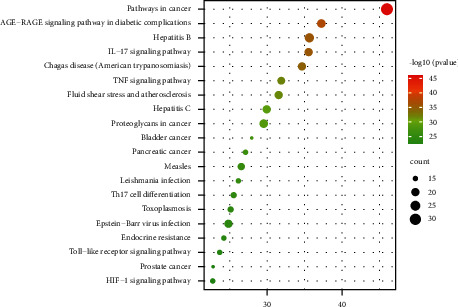
KRGG analysis of core targets.

The GO and KEGG analysis results of 57 core targets after the arithmetic by Metascape are shown in Figures 5 and 6.

GO analysis (*P* < 0.05) manifested that 1552 entries of biological processes were yielded, such as the apoptotic signaling pathway, blood vessel development, and response to inorganic substances. 34 entries of cell components were yielded, such as membrane raft, membrane microdomain, and transcription regulator complex. 94 entries of molecular functions were yielded, such as transcription factor binding, kinase binding, and protein kinase binding.

KEGG analysis (*P* < 0.05) showed that AR treatment of CRF involved 137 pathways. The top 5 were pathways in cancer, AGE-RAGE pathway, hepatitis B, IL-17 pathway, and Chagas disease.

### 3.5. Molecular Docking

The top five active compounds were selected to dock to the top five core targets in turn to calculate the docking scores (Table 4).

**Figure 7:**
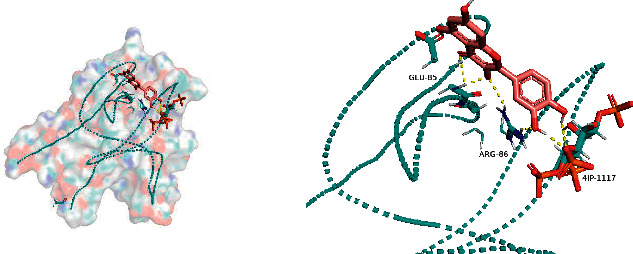
Docking of quercetin and AKT1.

**Figure 8:**
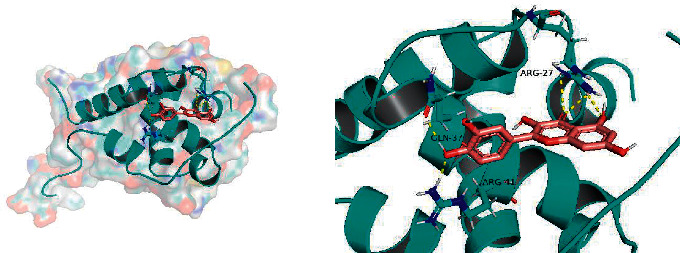
Docking of quercetin and TP53.

**Figure 9:**
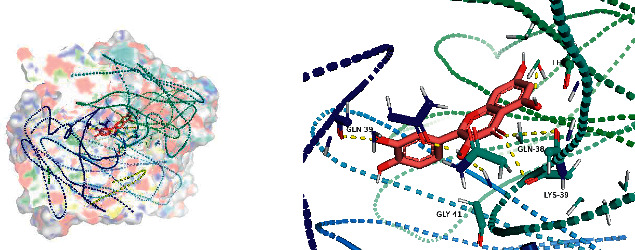
Docking of quercetin and VEGFA.

**Figure 10:**
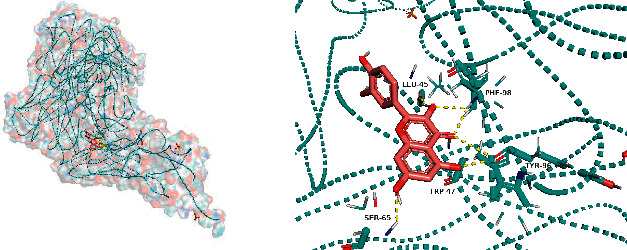
Docking of quercetin and IL-6.

**Figure 11:**
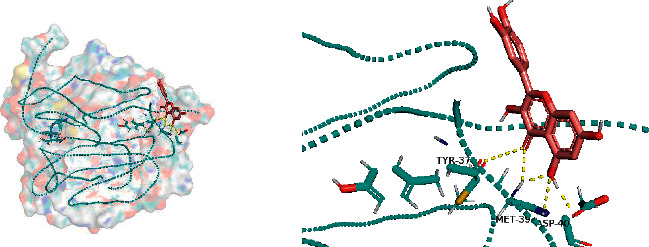
Docking of quercetin and CASP3.

All docking scores were <−5.0, and the abovementioned 5 active compounds docked well with the 5 core targets. Take quercetin as an example to show the visual pictures of its docking with 5 core targets, as shown in Figures 7–11.

## 4. Discussion

Assessment of AC-T network demonstrated that the top five active compounds in the treatment of CRF by AR were quercetin, kaempferol, 7-O-methylisomucronulatol, formononetin, and isorhamnetin.

Quercetin exerts its antifatigue effect mainly by eliminating metabolic accumulation, and as an effective antioxidant, it has the ability to scavenge free radicals while regulating mitochondrial biogenesis [20]. Studies have shown that quercetin can change the energy metabolism of mice and effectively improve their physical endurance [21]. Quercetin can also exert antitumor activity by inducing apoptosis, inhibiting cell proliferation, and inhibiting angiogenesis and metastasis [22]. In addition, quercetin has pharmacological effects such as anti-inflammatory, antibacterial, antiviral, and immune regulation [23]. Many previous studies have proved that kaempferol has good anti-inflammatory, immune regulation, antioxidation, antitumor, and other pharmacological properties [20]. It has been shown that kaempferol can induce Th1-mediated immune responses through dendritic cells [24]. Kaempferol can also exhibit antioxidant activities by increasing the expression of related antioxidant enzymes and reducing oxidative stress [25]. 7-O-Methylismucronulatol is a unique active component of AR and related research is rare at present. Formononetin is able to scavenge DPPH radicals in vitro and can promote the release of IL-4 to achieve the purpose of improving fatigue [26]. In addition, formononetin can also regulate Bcl-2, caspase-3, and other signaling pathways, inhibit tumor cell proliferation, and prevent tumor cell invasion [27]. Isorhamnetin is a powerful antioxidant, and its antioxidant effect also contributes to downregulating the expression of COX-2 in inflammatory responses [28]. It has been shown that isorhamnetin reduces the production of IL-12 and TNF-*α* and elevates heme oxygenase 1 protein levels to exert anti-inflammatory properties [29, 30]. Furthermore, isorhamnetin has antitumor effects and is extensively used in treating various tumors [31–33].

According to the attribute parameters of the core targets, AR mainly treats CRF through AKT1, TP53, VEGFA, IL-6, CASP3, and other targets.

AKT1 is one of the important isoforms of AKT. As a key signal downstream of PI3K, AKT mainly promotes cell growth, cell proliferation, and cell motility through the phosphorylation of a series of substrates [34]. The absence of AKT1 increases energy expenditure [35]. Liu et al. found that by upregulating the expression of the AKT1 gene, it can enhance the body's ability and exert anti-fatigue effects [36]. TP53 is the most commonly mutated gene in human cancer. The mutated TP53 is able to interfere with the recruitment and activity of T cells and myeloid cells and can lead to the loss of the cancer-suppressive function of wild-type TP53 [37]. TP53 controls inflammatory responses and regulates immunity and metabolism by regulating a variety of downstream target genes or molecules [38]. VEGFA is a key regulator of vascular growth. Landi et al. found that circulating levels of VEGFA were substantially decreased in patients with prolonged fatigue and that VEGFA was able to promote immunomodulators into the blood-brain barrier [39, 40]. In addition, VEGFA is an important target for cancer therapy, and VEGF inhibitors such as bevacizumab are now widely used. IL-6 plays a significant part in inflammation and immune responses. Inagaki et al. found that increased fatigue in cancer patients was associated with increased IL-6 levels [41]. IL-6 can induce T helper cell activation and keep the balance of regulatory T cells and Th17 cells [42]. Moreover, IL-6 may lead to abnormalities in the hypothalamic-pituitary-adrenal axis [43]. CASP3 is the main executor of apoptosis and is associated with DNA fragmentation, chromatin condensation, and apoptosome formation. Existing studies have shown that CASP3 gene polymorphisms are associated with cancer risk [44].

KEGG analysis of core targets showed that AR treatment of CRF involved a variety of signaling pathways related to tumors: pathways in cancer, AGE-RAGE, IL-17, TNF, Toll-like receptor, and HIF-1 pathways.

In the AGE-RAGE signaling pathway, AGEs interact through AGE-RAGE to bind and trigger RAGEs that play a key role in inflammation and oxidative stress [45]. Furthermore, AGE-RAGE acts synergistically to affect programmed cell death signaling and promote cancer development [46]. In the IL-17 signaling pathway, IL-17 is a pleiotropic proinflammatory cytokine involved in various processes such as host defense, inflammatory diseases, tissue repair, and cancer development [47]. Studies have shown that IL-17 may promote cancer development by promoting chronic tissue inflammation and can prevent cancer cells from receiving immune surveillance [48]. In the TNF signaling pathway, as one of the important inflammatory mediators, TNF-*α* regulates immunity, host defense, and inflammation by regulating cytokine production, cell survival, and cell death [49]. Moss et al. found that in patients with fatigue, TNF-*α* dramatically multiplied [50]. In addition, present studies have proved that TNF-*α* is involved in regulating and interfering with energy metabolism [51]. In the Toll-like receptor signaling pathway, activation of TLRs may underlie the pathophysiology of multiple diseases including chronic fatigue syndrome, autoimmune diseases, and depression [52]. TLRs participate in the inchoate activation of immune response by recognizing host-derived macromolecules [53]. In addition, in different tumor types, the expression of TLRs may lead to tumor progression or regression [54]. In the HIF-1 signaling pathway, HIF-1 has the ability to regulate the related genes of energy metabolism and is involved in cellular accommodation to low oxygen [55]. Furthermore, the HIF-1 signaling pathway participates in key processes in various tumor biologies such as cell proliferation and apoptosis, immune response, and invasion and metastasis [56].

## 5. Conclusion

In summary, quercetin, kaempferol, 7-O-methylisomucronulatol, formononetin, isorhamnetin, and other active compounds in AR may exert positive effects via AKT1, TP53, VEGFA, IL6, CASP3, and other targets, and through AGE-RAGE, IL-17, TNF, Toll-like receptor, and HIF-1 signaling pathways in treating CRF.

## Figures and Tables

**Table 1:** Active compounds of AR.

Number	Molecule ID	Molecule name	OB (%)	DL
AR1	MOL000378	7-O-Methylisomucronulatol	74.69	0.3
AR2	MOL000392	Formononetin	69.67	0.21
AR3	MOL000433	FA	68.96	0.71
AR4	MOL000380	(6aR, 11aR)-9, 10-Dimethoxy-6a, 11a-dihydro-6H-benzofurano [3, 2-c] chromen-3-ol	64.26	0.42
AR5	MOL000211	Mairin	55.38	0.78
AR6	MOL000371	3, 9-Di-O-methylnissolin	53.74	0.48
AR7	MOL000239	Jaranol	50.83	0.29
AR8	MOL000354	Isorhamnetin	49.6	0.31
AR9	MOL000417	Calycosin	47.75	0.24
AR10	MOL000098	Quercetin	46.43	0.28
AR11	MOL000422	Kaempferol	41.88	0.24
AR12	MOL000442	1, 7-Dihydroxy-3, 9-dimethoxy pterocarpene	39.05	0.48
AR13	MOL000296	Hederagenin	36.91	0.75
AR14	MOL000379	9, 10-Dimethoxypterocarpan-3-O-*β*-D-glucoside	36.74	0.92
AR15	MOL000033	(3S,8S,9S,10R,13R,14S,17R)-10,13-Dimethyl-17-[(2R,5S)-5-propan-2-yloctan-2-yl]-2,3,4,7,8,9,11,12,14,15,16,17-dodecahydro-1H-cyclopenta[a]phenanthren-3-ol	36.23	0.78
AR16	MOL000387	Bifendate	31.1	0.67

**Table 2:** The top five active compounds of AR.

Number	Molecule name	BC	CC	DC
AR10	Quercetin	0.745	0.613	144
AR11	Kaempferol	0.157	0.414	57
AR1	7-O-Methylisomucronulatol	0.119	0.388	40
AR2	Formononetin	0.106	0.380	35
AR8	Isorhamnetin	0.064	0.376	31

**Table 3:** The top fifteen core targets.

Number	Gene name	BC	CC	DC
1	AKT1	0.056	0.844	106
2	TP53	0.049	0.823	103
3	VEGFA	0.029	0.788	97
4	IL-6	0.031	0.778	94
5	CASP3	0.019	0.756	91
6	JUN	0.017	0.756	89
7	TNF	0.020	0.747	88
8	MYC	0.020	0.751	88
9	MAPK1	0.024	0.743	86
10	EGFR	0.023	0.739	84
11	MAPK8	0.014	0.730	82
12	EGF	0.022	0.722	81
13	PTGS2	0.014	0.722	81
14	ESR1	0.023	0.722	81
15	CCND1	0.013	0.710	80

**Table 4:** Docking score.

	AKT1	TP53	VEGFA	IL-6	CASP3
Quercetin	−5.9	−5.7	−7.5	−8.0	−7.5
Kaempferol	−5.9	−6.2	−7.6	−7.9	−7.8
7-O-Methylisomucronulatol	−5.6	−6.1	−6.8	−6.6	−6.8
Formononetin	−6.5	−6.6	−7.3	−7.8	−7.9
Isorhamnetin	−5.8	−5.9	−7.6	−8.0	−7.5

## Data Availability

The data used to support this research can be obtained from the corresponding author upon reasonable request.

## Abbreviations

AC-T: Active compound-target
AR: *Astragali Radix*
BC: Betweenness centrality
CC: Closeness centrality
CRF: Cancer-related fatigue
DC: Degree centrality
DL: Drug likeness
OB: Oral bioavailability
PPI: Protein-protein interaction.
